# Microglia mediate the increase in slow-wave sleep associated with high ambient temperature

**DOI:** 10.1186/s12576-024-00929-0

**Published:** 2024-07-17

**Authors:** Sena Hatori, Futaba Matsui, Zhiwen Zhou, Hiroaki Norimoto

**Affiliations:** 1 Graduate School of Science, Nagoya University, Nagoya, 464-8602 Japan; 2 Graduate School of Medicine, Hokkaido University, Sapporo, 060-8638 Japan

**Keywords:** Sleep, Microglia, Temperature

## Abstract

An increase in ambient temperature leads to an increase in sleep. However, the mechanisms behind this phenomenon remain unknown. This study aimed to investigate the role of microglia in the increase of sleep caused by high ambient temperature. We confirmed that at 35 °C, slow-wave sleep was significantly increased relative to those observed at 25 °C. Notably, this effect was abolished upon treatment with PLX3397, a CSF1R inhibitor that can deplete microglia, while sleep amount at 25 °C was unaffected. These observations suggest that microglia play a pivotal role in modulating the homeostatic regulation of sleep in response to the fluctuations in ambient temperature.

## Introduction

Sleep is affected by several external factors including light, sound, and ambient temperature. Various reports have been made on the relationship between sleep and ambient temperature. For example, it has been reported that elevating ambient temperature increases the depth of sleep [1] and the amount of slow-wave sleep (SWS) [2–4]. However, the detailed mechanism underlying these changes has yet to be elucidated.

Microglia play a role in maintaining homeostasis through synaptic remodeling, release of cytokines, and phagocytosis [5]. Several studies have investigated the relationship between microglia and sleep, including a report indicating that depletion of microglia suppresses the rebound of SWS after sleep deprivation [6]. This suggests a role for microglia in sleep homeostasis.

In this study, we hypothesized that microglia may be involved in the increased sleep amount caused by high ambient temperature. To test this hypothesis, we used the colony-stimulating factor 1 receptor (CSF1R) inhibitor PLX3397 to deplete microglia in the mouse brain and performed electrophysiological recordings at two different temperatures (25 °C and 35 °C) in both the control group mice and the microglia depleted mice.

## Materials and methods

### Animals

All procedures involving the use of animals complied with the guidelines of the National Institutes of Health and were approved by the Animal Care and Use Committee of the Hokkaido University (Approval numbers: 21-0092).

Wild-type C57BL/6J mice (6-to-9 weeks old; SLC, Shizuoka, Japan) were housed under 12-h light/dark cycle. Food and water were given ad libitum. All efforts were made to minimize the animals' suffering and the number of animals used.

### PLX3397 treatment

PLX3397 (C-1271; Chemgood, VA, USA) was mixed into standard rodent chow (Research Diets Inc., NJ, USA) at the concentration of 290 mg/kg to achieve a dose of ~ 46 mg/kg body weight per day for more than 3 weeks. This is an effective way to observe the role of microglia in animals’ natural behavior [7].

### Surgery

Mice were anesthetized with isoflurane and placed in a stereotaxic apparatus. During the surgery, mice were exposed to 1.5–2% isoflurane and kept on a warm pad to prevent them from lowering their body temperature. Electroencephalogram (EEG) electrodes (stainless steel screws; Matsumoto, Chiba, Japan) were implanted epidurally over the parietal area (AP = −1 mm, ML = 1.5 mm). Electromyogram (EMG) electrodes (stainless steel wires, A-M systems, WA, USA) were implanted into the neck muscle. After the proper suturing, mice were kept on the warm pad for recovery and then transferred to the home cages.

### EEG/EMG recording

After an adequate recovery period, each mouse was placed into an acrylic box with an open top (30 × 30 × 30 cm) in a soundproof chamber. Following cage habituation for one day, EEG and EMG at 25 °C were recorded for 8 h, starting from the onset of the light phase. Then, the temperature was set to 35 °C. Recordings at 35 °C were conducted three days later on the same schedule as at 25 °C. Recordings were acquired using an acquisition board (Open Ephys, GA, USA) and 32Ch headstages (Intan Technologies, CA, USA). Signals were sampled at 1 kHz with a 0.1–100 Hz band pass filter.

### Sleep analysis

All analyses were conducted using MATLAB (R2022a; MathWorks Inc., MA, United States). The EEG data underwent fast Fourier transform (FFT) analysis with a 10-s window and 1-s step. The EMG data were band-pass filtered between 20–100 Hz, and the root mean square (RMS) was calculated using a 10-s window (EMG envelope). Subsequently, the EEG and RMS of EMG data were segmented into 4-s epochs. To determine vigilance states, a decision tree–based algorithm was employed based on delta (δ) power (0.1–3 Hz), theta (θ) ratio (θ band (6–9 Hz) power/all band (0.1–30 Hz) power), and EMG envelope. Following this analysis, epochs identified as a different vigilance state from the surrounding epochs are adjusted to match the state of the previous epoch. Additionally, epochs judged as rapid eye movement sleep (REMS) directly from wakefulness were classified as wakefulness. This series of methods resembles the algorithm of SleepSign (Kissei Comtec, Nagano, Japan), which is one of the most conventional methods for determining vigilance states [8].

### Immunohistochemistry

Animals were perfused transcardially with cold phosphate-buffered saline (PBS) followed by 4% paraformaldehyde (PFA) in phosphate buffer. The brains were quickly removed, placed in 4% PFA for 24 h, and then placed in PBS with 30% sucrose for 48 h at 4 °C. 100-μm-thick brain sections were made using a sliding microtome (HM450, epredia, NH, United States) at −21 °C.

The brain sections were permeabilized, and nonspecific staining was blocked with 10% goat serum (Vector Laboratory, California, USA) in 0.3% Triton X-100 (TaKaRa, Shiga, Japan) in PBS. For immunohistochemical detection of IBA1, we used the primary antibody rabbit anti-IBA1 antibody (1:500, Wako, Osaka, Japan). Sections were incubated overnight at 4 °C with primary antibody. After extensive washes in PBS, the sections were incubated with the appropriate secondary antibody conjugated with Alexa Fluor dyes (1:500; Invitrogen, MA, USA). After incubation overnight at 4 °C with the secondary antibody, the sections were extensively washed with PBS, incubated with Hoechst for nuclear staining, and mounted on glass slides.

The sections were photographed using an FV1000 confocal scanning microscope with 10× / 20× objective lenses (Olympus, Tokyo, Japan) and BZ-X810 microscope with 4× objective lenses (Keyence, Osaka, Japan). Images were processed using Fiji ImageJ (NIH, USA) and BZ-II analysis application.

## Results

### The effect of high ambient temperature on sleep

**Fig. 1:**
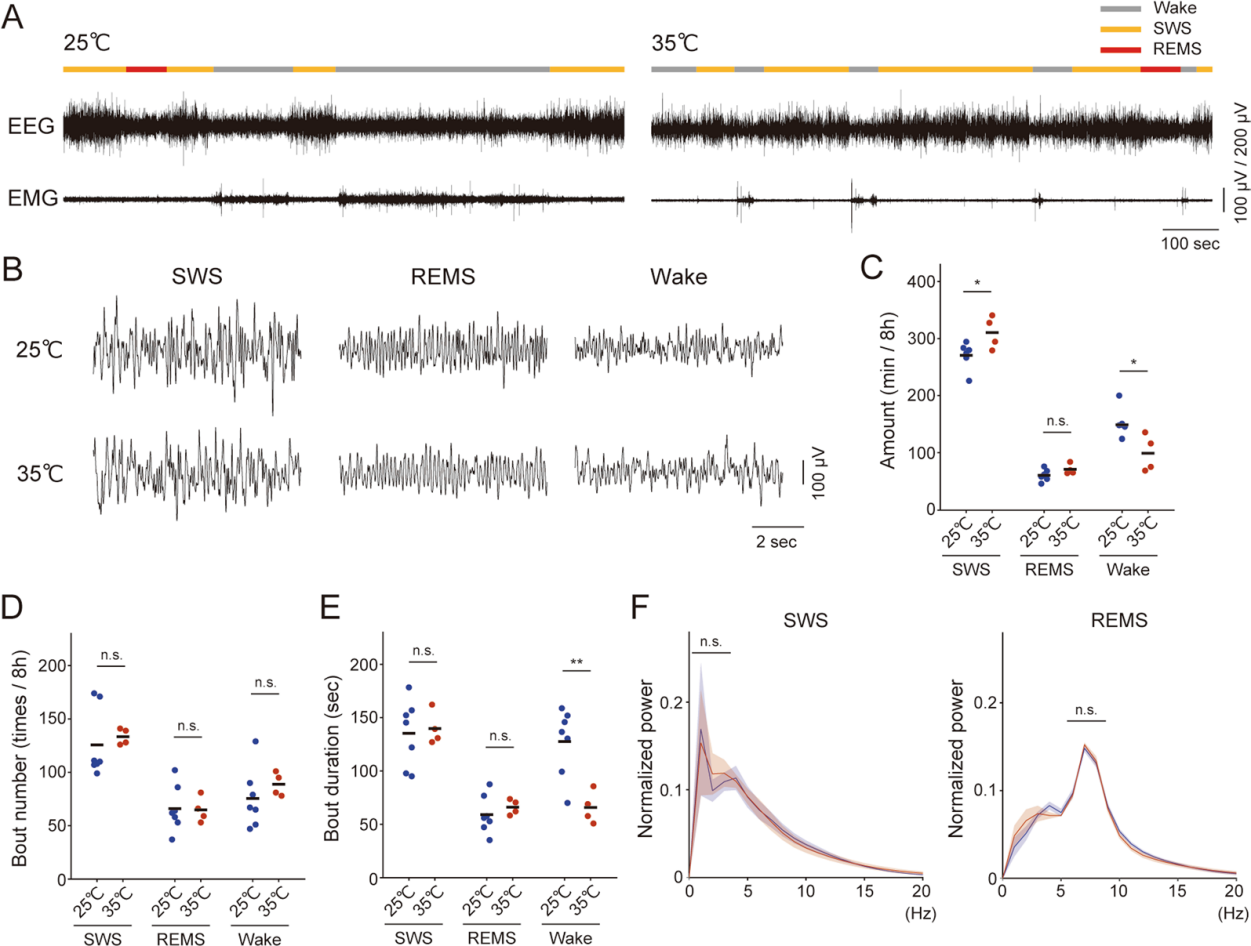
| SWS increases at high ambient temperature. **A** Classification of vigilance states at each temperature. The two bottom plots indicate the EEG and EMG, which were used to define vigilance states, i.e., SWS, REMS, and wakefulness (see “Methods”). **B** Examples of EEG traces in each vigilance state (top, 25 °C; bottom, 35 °C). **C** Comparison of the amount of SWS, REMS, and wake between 25 °C and 35 °C conditions in an 8-h recording. *P < 0.05. Each dot represents an individual, and the black bar shows the mean of the group. **D** Comparison of bout numbers of SWS, REMS, and wake between 25 °C and 35 °C conditions in an 8-h recording. Each dot represents an individual, and the black bar shows the mean of the group. **E** Comparison of bout duration of SWS, REMS, and wake between 25 °C and 35 °C conditions. Each dot represents an individual, and the black bar indicates the mean of the group. **F** Comparison of power spectrum between 25 °C and 35 °C conditions during SWS (left) and REMS (right). The normalized EEG power in each frequency bin is expressed as a proportion of the total EEG power. The data are presented as mean ± SEM

First, we investigated the effect of ambient temperature on sleep amount. EEG and EMG were recorded from ZT0 to ZT8 at 25 °C. Subsequently, the temperature was set to 35 °C and recording were made following three days of habituation at 35 °C. Vigilance states were determined based on EEG and EMG signals (Fig. 1A, B). The total amount of SWS showed a significant increase at 35 °C, while the amount of wakefulness decreased (Fig. 1C, SWS: *P* = 0.028,* t*_9_ = −2.6, Student’s *t*-test; REMS: *P* = 0.12, *t*_9_ = −1.7, Student’s *t*-test; Wake: *P* = 0.019, *t*_9_ = 2.9, Student’s *t*-test). Further analysis revealed no significant differences in the number of bouts of SWS, REMS, or wake episodes (Fig. 1D, SWS: *P* = 0.65,* t*_9_ = −0.47, Student’s *t*-test; REMS: *P* = 0.92, *t*_9_ = 0.11, Student’s *t*-test; Wake: *P* = 0.39, *t*_9_ = −0.90, Student’s *t*-test), whereas the duration of wake bouts was significantly decreased at 35 °C (Fig. 1E, SWS: *P* = 0.80,* t*_9_ = −0.26, Student’s *t*-test; REMS: *P* = 0.47, *t*_9_ = −0.75, Student’s *t*-test; Wake: *P* = 0.0059, *t*_9_ = 3.6, Student’s *t*-test). No significant differences in EEG power were observed under these conditions in each sleep stage (Fig. 1F, δ power in SWS: *P* = 0.93, *t*_9_ = − 0.087, Student’s *t*-test; θ power in REMS: *P* = 0.93, *t*_9_ = −0.093, Student’s *t*-test).

### Microglial depletion impairs sleep homeostasis in response to high ambient temperature

**Fig. 2:**
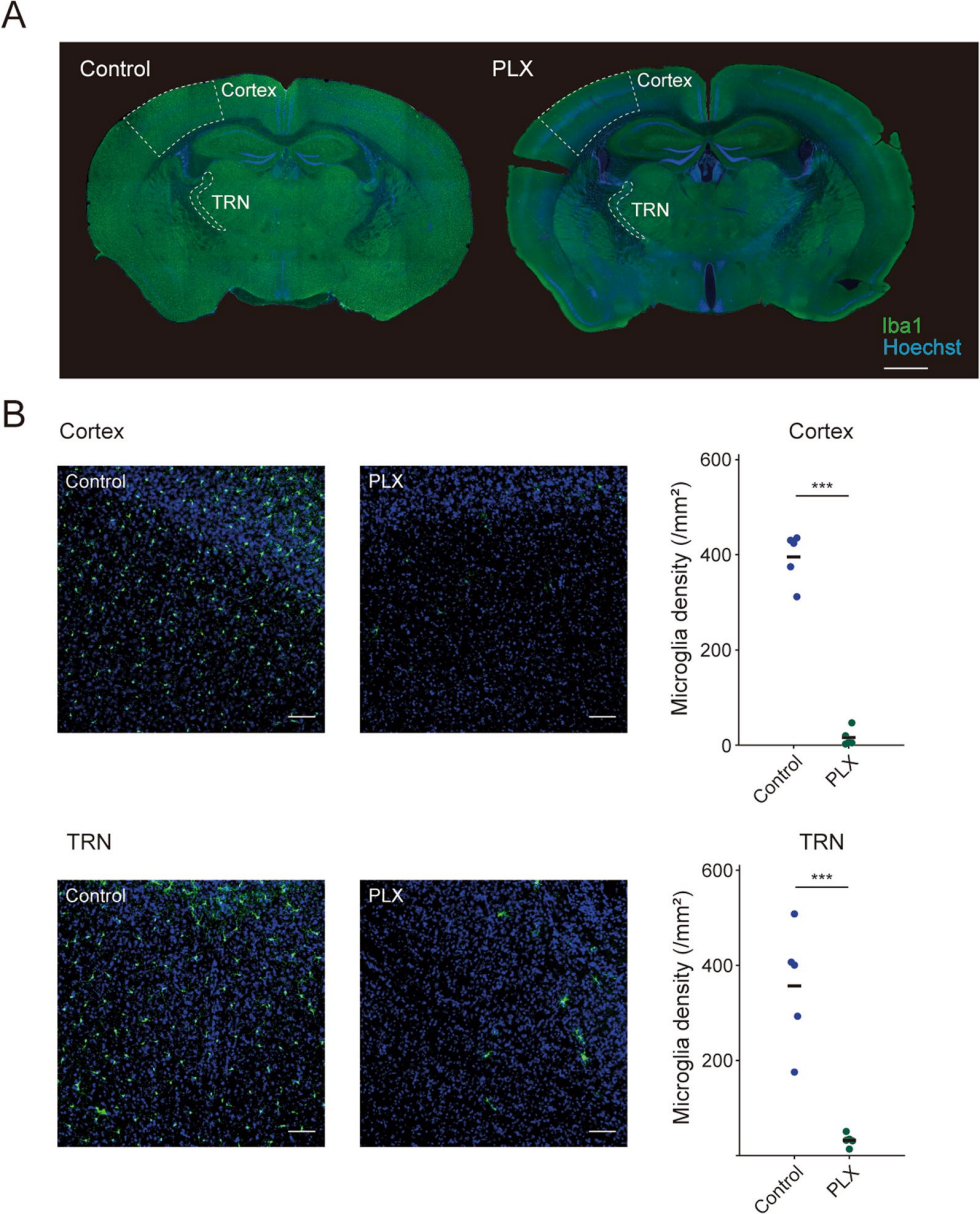
| The CSF1R inhibitor PLX3397 ablates most microglia. **A** Whole brain sections from a control mouse and PLX3397 treated mouse (PLX) with Hoechst staining (blue) and immunolabeling for the microglial marker IBA1 (green). The cortex and thalamic reticular nucleus (TRN) in which microglia density is quantified in **B** are marked with white dotted lines. **B** Left, enlarged images in the cortex (top) and TRN (bottom). Right, microglia density is quantified. ***P < 0.001. Each dot represents an individual, and black bar shows the mean of the group

Next, to examine the role of microglia, we conducted recordings from mice treated with PLX3397, an inhibitor of CSF1R. Since the CSF1R signal is essential for the survival of microglia in the brain, inhibition of this pathway results in microglial removal. Previous reports have shown that continuous administration of PLX3397 for three weeks almost completely depletes microglia in the brain [9]. In this study, we confirmed that via PLX3397 administration for more than three weeks, over 95% of microglia were depleted in the entire brain (Fig. 2A) including the cerebral cortex and thalamic reticular nucleus (TRN), which are necessary for generating sleep oscillations during SWS (Fig. 2B, Cortex: *P* = 3.5 × 10^–7^, *t*_8_ = 15, Student’s *t*-test; TRN: *P* = 4.6 × 10^–4^, *t*_8_ = 5.7, Student’s *t*-test).

**Fig. 3:**
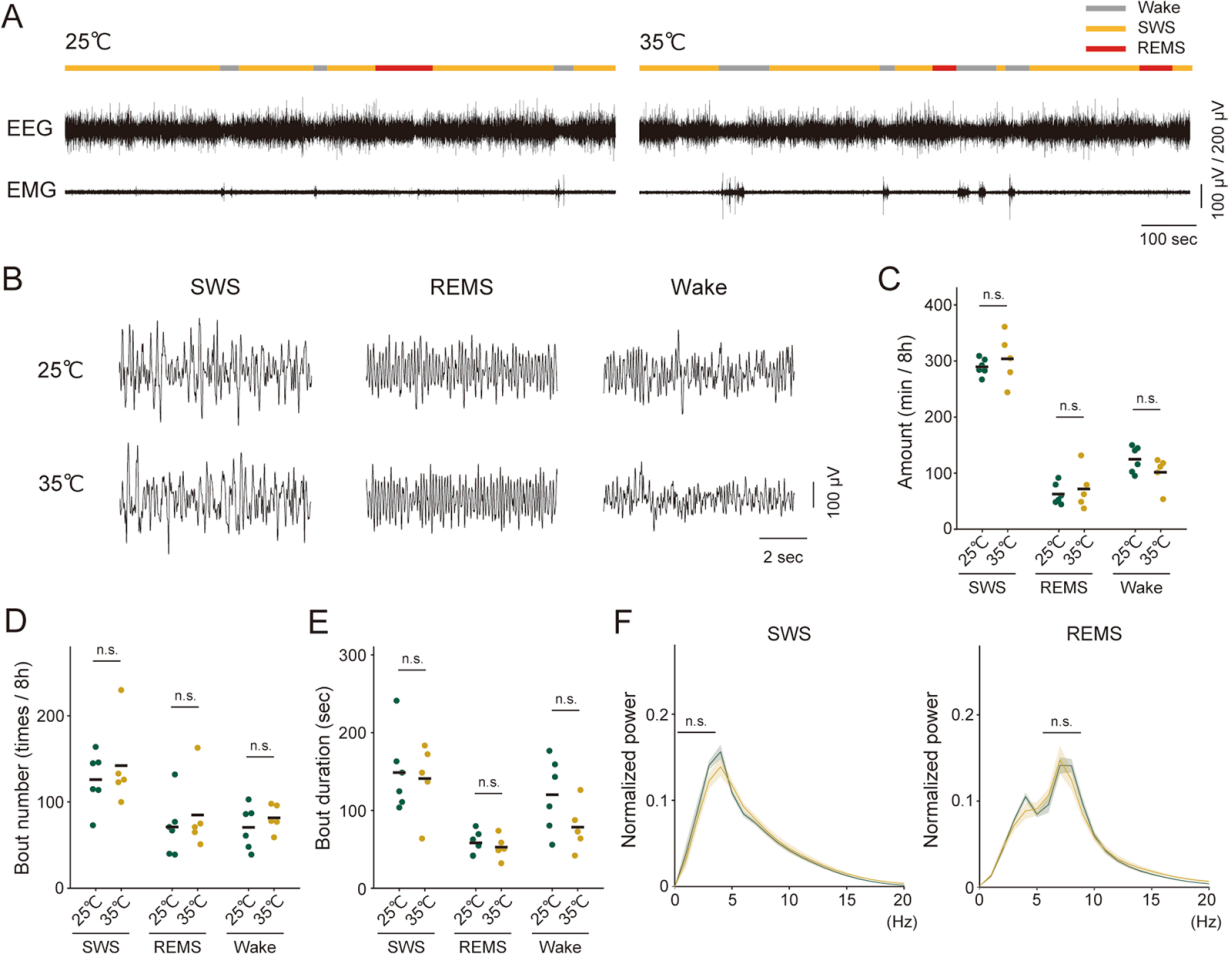
| Ablation of microglia impairs the increase in sleep duration induced by high ambient temperature. **A** Classification of vigilance states at each temperature in PLX3397 treated mice. The two bottom plots indicate the EEG and EMG, which were used to define vigilance states, i.e., SWS, REMS, and wakefulness (see “Methods”). **B** Examples of EEG traces in each vigilance state. **C** Comparison of SWS, REMS, and wake amounts between 25 °C and 35 °C conditions in an 8-h recording. Each dot represents an individual, and black bar shows the mean of the group. **D** Comparison of bout numbers of SWS, REMS, and wake between 25 °C and 35 °C conditions in 8-h recording. Each dot represents an individual, and black bar shows the mean of the group. **E** Comparison of bout duration of SWS, REMS, and wake between 25 °C and 35 °C conditions. Each dot represents an individual, and black bar shows the mean of the group. **F** Comparison of the power spectrum between 25 °C and 35 °C conditions during SWS (left) and REMS (right). The normalized EEG power in each frequency bin is expressed as a proportion of the total EEG power. The data are presented as mean ± SEM

At 25 °C, there was no difference in the duration of wakefulness or sleep between the control group and PLX3397 treated group (SWS: *P* = 0.088,* t*_11_ = − 1.9, Student’s *t*-test; REMS: *P* = 0.69, *t*_11_ = −0.40, Student’s *t*-test; Wake: *P* = 0.12, *t*_11_ = 1.7, Student’s *t*-test), indicating microglia do not affect sleep/wake amount under light on condition. When the temperature was raised to 35 °C, in contrast to the mice without PLX3397 treatment, the PLX3397 treated group did not show an increase in sleep amount (Fig. 3C, SWS: *P* = 0.49, *t*_9_ = −0.73, Student’s *t*-test; REMS: *P* = 0.61, *t*_9_ = −0.53, Student’s *t*-test; Wake: *P* = 0.17, *t*_9_ = 1.5, Student’s *t*-test). Also, after PLX treatment, there was no significant difference between 25 °C and 35 °C in bout number (Fig. 3D, SWS: *P* = 0.53, *t*_9_ = −0.64, Student’s *t*-test; REMS: *P* = 0.57, *t*_9_ = −0.59, Student’s *t*-test; Wake: *P* = 0.42, *t*_9_ = −0.84, Student’s *t*-test) or bout duration of SWS, REMS or wake (Fig. 3E, SWS: *P* = 0.80, *t*_9_ = 0.26, Student’s *t*-test; REMS: *P* = 0.56, *t*_9_ = 0.61, Student’s *t*-test; Wake: *P* = 0.13, *t*_9_ = 1.7, Student’s *t*-test). Similar to the control group mice, there was no difference between the 25 °C and 35 °C conditions in the power spectra of EEG both during SWS and REMS in microglia-depleted mice (Fig. 3F, δ power in SWS: *P* = 0.24, *t*_9_ = 1.3, Student’s *t*-test; θ power in REMS: *P* = 0.77, *t*_9_ = 0.30, Student’s *t*-test). These findings suggest that microglia are involved in the homeostatic regulation of sleep amount in response to an increase in ambient temperature.

## Conclusion

In this study, we found that raising ambient temperature led to an increase in the amount of SWS, and that this effect was abolished by microglia depletion. Also, we have shown that microglial depletion induces no changes in sleep amount during the light period, which is consistent with previous studies [10, 11]. Wisor et al. showed that following sleep deprivation, the rebound increase of δ power in SWS, a reliable marker of sleep need, is abolished by the inactivation of microglia induced by minocycline, indicating a direct role of microglia in the response to sleep loss [6]. These results indicate that while microglia are not involved in the regulation of baseline sleep during the light period, they do play a role in maintaining sleep homeostasis.

Given the fact that ambient temperature does not affect body temperature significantly [12], it is possible that peripheral areas detect changes in ambient temperature, relay this information to the brain regions including the preoptic area [13], and activate microglia. Activated microglia can release inflammatory cytokines such as IL-1β and TNFα, which have been shown to increase the amount of SWS [14, 15]. Recently, a study has also shown that the increase of intracellular Ca^2+^ in microglia facilitates sleep [16]. Future studies with in vivo real-time imaging and manipulation of microglia will help clarify the mechanisms.

In conclusion, increased SWS amount induced by high ambient temperature may be a result of microglial functions. Intervening in the activity of microglia could be a target to rescue sleep/wake disturbances associated with high ambient temperature.

## Data Availability

The data sets used and/or analyzed during the current study are available from the corresponding author on reasonable request.

## References

[1] Ajwad A, Huffman D, Yaghouby F, OrHara BF, Sunderam S (2018). Sleep depth enhancement through ambient temperature manipulation in mice. Annu Int Conf IEEE Eng Med Biol Soc.

[2] Roussel B, Turrillot P, Kitahama K (1984). Effect of ambient temperature on the sleep-waking cycle in two strains of mice. Brain Res.

[3] Li L, Zhang MQ, Sun X, Liu WY, Huang ZL, Wang YQ (2022). Role of dorsomedial hypothalamus GABAergic neurons in sleep-wake states in response to changes in ambient temperature in mice. Int J Mol Sci.

[4] Jhaveri KA, Trammell RA, Toth LA (2007). Effect of environmental temperature on sleep, locomotor activity, core body temperature and immune responses of C57BL/6J mice. Brain Behav Immun.

[5] Tremblay ME, Stevens B, Sierra A, Wake H, Bessis A, Nimmerjahn A (2011). The role of microglia in the healthy brain. J Neurosci.

[6] Wisor JP, Schmidt MA, Clegern WC (2011). Evidence for neuroinflammatory and microglial changes in the cerebral response to sleep loss. Sleep.

[7] Green KN, Crapser JD, Hohsfield LA (2020). To kill a microglia: a case for CSF1R inhibitors. Trends Immunol.

[8] Pinzar E, Kanaoka Y, Inui T, Eguchi N, Urade Y, Hayaishi O (2000). Prostaglandin D synthase gene is involved in the regulation of non-rapid eye movement sleep. Proc Natl Acad Sci USA.

[9] Matsui F, Yamaguchi ST, Kobayashi R, Ito S, Nagashima S, Zhou Z, Norimoto H (2023). Ablation of microglia does not alter circadian rhythm of locomotor activity. Mol Brain.

[10] Corsi G, Picard K, di Castro MA, Garofalo S, Tucci F, Chece G, Del Percio C, Golia MT, Raspa M, Scavizzi F (2022). Microglia modulate hippocampal synaptic transmission and sleep duration along the light/dark cycle. Glia.

[11] Liu H, Wang X, Chen L, Chen L, Tsirka SE, Ge S, Xiong Q (2021). Microglia modulate stable wakefulness via the thalamic reticular nucleus in mice. Nat Commun.

[12] Russell LN, Hyatt WS, Gannon BM, Simecka CM, Randolph MM, Fantegrossi WE (2021). Effects of laboratory housing conditions on core temperature and locomotor activity in mice. J Am Assoc Lab Anim Sci.

[13] Rothhaas R, Chung S (2021). Role of the preoptic area in sleep and thermoregulation. Front Neurosci.

[14] Zielinski MR, Gibbons AJ (2022). Neuroinflammation, sleep, and circadian rhythms. Front Cell Infect Microbiol.

[15] Krueger JM (2008). The role of cytokines in sleep regulation. Curr Pharm Des.

[16] Ma C, Li B, Silverman D, Ding X, Li A, Xiao C, Huang G, Worden K, Muroy S, Chen W (2024). Microglia regulate sleep through calcium-dependent modulation of norepinephrine transmission. Nat Neurosci.

